# The Assessment of Multiplex PCR in Identifying Bacterial Infections in Patients Hospitalized with SARS-CoV-2 Infection: A Systematic Review

**DOI:** 10.3390/antibiotics12030465

**Published:** 2023-02-24

**Authors:** Iulia Bogdan, Tejaswi Gadela, Felix Bratosin, Catalin Dumitru, Alin Popescu, Florin George Horhat, Rodica Anamaria Negrean, Razvan Mihai Horhat, Ion Cristian Mot, Adrian Vasile Bota, Carmen Nicoleta Stoica, Bogdan Feciche, Andrei Nicolae Csep, Roxana Manuela Fericean, Gratiana Nicoleta Chicin, Iosif Marincu

**Affiliations:** 1 Department XIII, Discipline of Infectious Diseases, “Victor Babes” University of Medicine and Pharmacy Timisoara, Eftimie Murgu Square 2, 300041 Timisoara, Romania; 2 Doctoral School, “Victor Babes” University of Medicine and Pharmacy Timisoara, Eftimie Murgu Square 2, 300041 Timisoara, Romania; 3 Methodological and Infectious Diseases Research Center, Department of Infectious Diseases, “Victor Babes” University of Medicine and Pharmacy, Eftimie Murgu Square 2, 300041 Timisoara, Romania; 4 School of General Medicine, Bhaskar Medical College, Amdapur Road 156-162, Hyderabad 500075, India; 5 Department of Obstetrics and Gynecology, “Victor Babes” University of Medicine and Pharmacy Timisoara, Eftimie Murgu Square 2, 300041 Timisoara, Romania; 6 Multidisciplinary Research Center on Antimicrobial Resistance (MULTI-REZ), Microbiology Department, “Victor Babes” University of Medicine and Pharmacy, 300041 Timisoara, Romania; 7 Faculty of Medicine and Pharmacy, University of Oradea, 410073 Oradea, Romania; 8 Department of Conservative Dentistry and Endodontics, Faculty of Dental Medicine, “Victor Babes” University of Medicine and Pharmacy Timisoara, Eftimie Murgu Square 2, 300041 Timisoara, Romania; 9 ENT Department, “Victor Babes” University of Medicine and Pharmacy Timisoara, 2 Eftimie Murgu Sq, 300041 Timisoara, Romania; 10 Oradea Emergency Clinical Hospital, Infectious Diseases Department, 410087 Oradea, Romania; 11 Department of Urology, Satu-Mare County Emergency Hospital, Strada Ravensburg 2, 440192 Satu-Mare, Romania; 12 Department of Psycho-Neuroscience and Recovery, Faculty of Medicine and Pharmacy, University of Oradea, 410087 Oradea, Romania; 13 Faculty of General Medicine, “Vasile Goldis” Western University of Arad, Bulevardul Revolutiei 94, 310025 Arad, Romania; 14 National Institute of Public Health, Strada Doctor Leonte Anastasievici 1-3, 050463 Bucuresti, Romania

**Keywords:** multiplex PCR, bacterial infection, co-infection, COVID-19, SARS-CoV-2

## Abstract

Bacterial infection can occur in patients hospitalized with SARS-CoV-2 in various conditions, resulting in poorer outcomes, such as a higher death rate. This current systematic review was conducted in order to assess the efficiency of multiplex PCR in detecting bacterial infections in hospitalized COVID-19 patients, as well as to analyze the most common bacterial pathogens and other factors that interfere with this diagnosis. The research was conducted using four electronic databases (PubMed, Taylor&Francis, Web of Science, and Wiley Online Library). Out of 290 studies, nine were included in the systematic review. The results supported the use of multiplex PCR in detecting bacteria, considering its high sensitivity and specificity rates. The most common bacterial pathogens found were *Klebsiella pneumoniae*, *Staphylococcus aureus*, *Pseudomonas aeruginosa*, *Streptococcus pneumoniae*, and *Haemophilus influenzae.* The median age at admission was 61.5 years, and the majority of patients were men (70.3%), out of a total of 1553 patients. The proportion of ICU admission was very high, with a pooled proportion of 52.6% over the analyzed studies, and an average duration of hospitalization of 13 days. The mortality rate was proportionally high, as was the rate of ICU admission, with a pooled mortality of 24.9%. It was discovered that 65.2% of all patients used antibiotics before admission, with or without medical prescription. Antibiotic treatment should be considered consciously, considering the high risks of developing antibiotic resistance.

## 1. Introduction

The severe acute respiratory syndrome coronavirus 2 (SARS-CoV-2) is the infectious agent that causes the condition known as coronavirus disease (COVID-19). Co-infections can lead to more severe consequences despite the fact that the majority of infected patients suffer mild to moderate symptoms that do not require any specific treatment [1,2,3]. The respiratory system is the primary organ that is targeted by the SARS-CoV-2 virus, which has a high rate of transmission from human to human [4,5,6]. In light of the fact that the virus is highly contagious, the presence of additional infections makes the clinical picture more complicated. This necessitates a multifaceted strategy and prompt treatment, as it was discovered that the majority of fatalities were due to the presence of more than one infection [7,8,9,10]. The most common bacterial infections, according to recent articles, systematic reviews and meta-analyses, are *Klebsiella pneumoniae*, *Streptococcus pneumoniae*, *Staphylococcus aureus*, *Mycoplasma pneumoniae*, *Chlamydophila pneumoniae*, *Legionella pneumophila*, and *Acinetobacter baumannii* [11,12,13,14]. These pathogens may be community-acquired, contracted outside of a hospital setting and diagnosed before or 48 h after admission, which indicates that the individual was infected prior or simultaneously to the SARS-CoV-2 infection. The same bacteria may also be a part of an underlying chronic infection; or they may be nosocomial, which indicates that they were contracted from a hospital setting and diagnosed at least 48 h after hospitalization for COVID-19 [15,16,17].

When it comes to the detection of pathogens, clinical microbiology has been an essential component. This is because the coronavirus outbreak required testing methods that were precise and carried out at a rapid speed [18,19,20,21]. Multiplex polymerase chain reaction, or multiplex PCR, is one of the testing procedures that can be used. This particular variation of PCR has shown promising results in a variety of DNA testing applications [22,23,24]. Multiplex polymerase chain reaction is a faster variation of the conventional polymerase chain reaction (PCR), and differs from it by amplifying more than one target sequence by employing multiple pairs of primers [25,26]. Real-time or reverse transcription PCR (RT-PCR) is also less time-consuming than the conventional PCR by having no post-amplification procedure and being able to detect genetic material from RNA as well. Antibiotics are still given to patients even though the treatment for COVID-19 does not involve the prescription of antibiotics. This is done in the hope of eliminating any potential bacterial infections that may be present, as the mortality rates are higher in patients who have a co-infection or a superinfection [27,28,29]. In spite of the fact that antibiotic use may be helpful, it is important to point out that there is a potential danger of developing antimicrobial resistance when using antibiotics; as a result, the choice of whether or not to receive antibiotic therapy ought to be considered carefully [30,31,32].

Since the methods of diagnosis and treatment decision-making can be affected by the presence of different bacteria in patients [11], we conducted this current systematic review in order to observe the clinical characteristics and outcomes in patients hospitalized due to COVID-19 with presenting bacterial co-infection or superinfection and the efficacy of a multiplex PCR in screening for bacterial infections.

## 2. Materials and Methods

The current systematic review search was conducted until January 2023 using four electronic databases (PubMed, Taylor&Francis, Web of Science, and Wiley Online Library). During our analysis of the studies, we took into consideration the setting in which the infection occurred, the nature of the pathogen, and the testing procedure. More specifically, the study cohort was required to have consisted of hospitalized patients who were infected with SARS-CoV-2, and the studies were required to involve screening for bacterial pathogens using the multiplex PCR method, regardless of the commercial variation. Different microbial detection panels were considered for inclusion (BioFire^®^ FilmArray^®^, BioFire^®^ Pneumonia Panel, and Superscript III). We took into consideration the sample size, clinical outcomes of hospitalized patients (such as admission at intensive unit care (ICU)), length of hospitalization, and the mortality rate, all of which comprise the negative outcomes that could occur; the most frequent bacteria encountered; the type of acquisition; and the overall result of multiplex PCR in detecting bacteria (sensitivity and specificity), or the result when compared to another type of screening.

**Figure 1:**
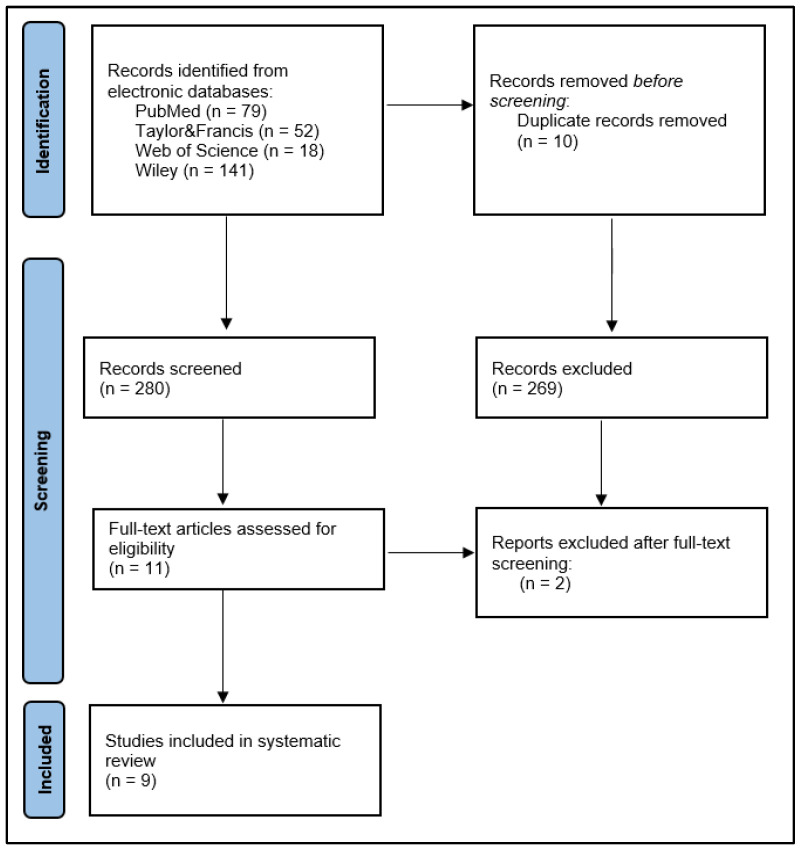
PRISMA flow diagram.

All information was gathered from the articles’ text, tables, figures, and online supplemental resources. The preliminary investigation yielded a total of 290 research publications. The search was constrained to only include academic papers that had been published after the year 2020. Although there were no restrictions regarding the language used, we only included articles that were written in English. Following the elimination of duplicates, we were left with a total of 280 articles to review. After screening the abstracts, a total of 269 records were excluded; as a result, 11 articles were subjected to full-text examination. Two further exclusions were made due to (1) a lack of data that was reported and (2) the use of a screening method other than multiplex PCR. As a result, this particular review of the literature consists of nine different study articles. Figure 1 provides a visual representation of the screening process. The search terms focused on the type of screening, the different kinds of pathogens, and the presence or absence of SARS-CoV-2 infection, more specifically “multiplex PCR” AND (“bacterial infections” OR nosocomial OR bacteria) AND (SARS-CoV-2 OR COVID-19). The systematic review was conducted in accordance with the Preferred Reporting Items for Systematic Reviews (PRISMA) [33] and PROSPERO protocol [34]. The current systematic review was registered to the Open-Science Framework (OSF) platform.

## 3. Results

### 3.1. Study Characteristics and Clinical Outcomes

Out of the nine studies included [35,36,37,38,39,40,41,42,43], most studies were retrospective [35,37,38,39,40,41], one article was cross-sectional [36], and two articles were prospective [42,43]. Regarding the place where the studies were conducted, there was a heterogeneous result since each study took place in another country, as follows: Saudi Arabia [35], Romania [36], Israel [37], Italy [38], Taiwan [39], Austria [40], France [41], Germany and Switzerland together [42], and Peru [43]. Regarding the sample size, almost half of the studies (44.4%) had a sample size of less than 100 patients [35,37,40,43], while the others (55.6%) ranged from 178 to 489 patients [38,39,41,42]. Reported data can be found in Table 1.

**Figure 2:**
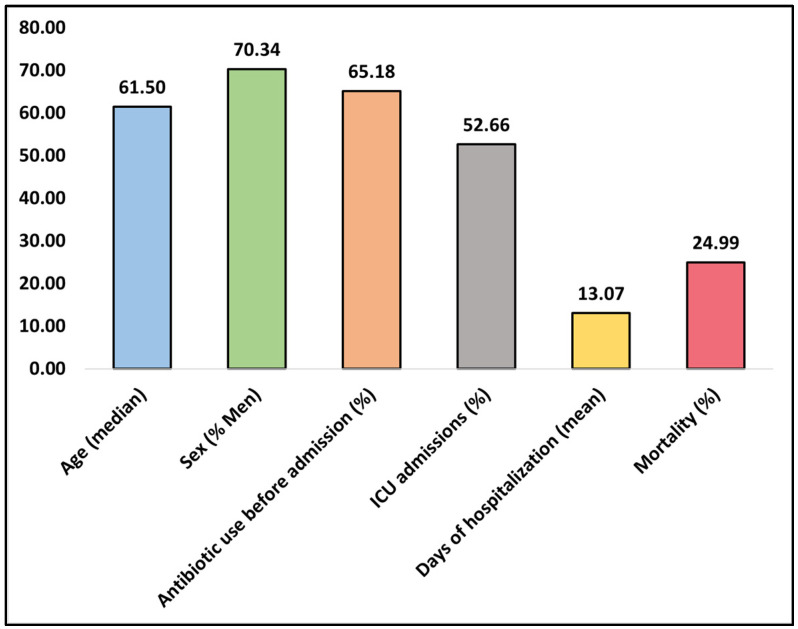
Summary of findings.

Patients who were hospitalized as a result of COVID-19 were included in the study’s cohort. These patients could have been treated in a standard unit or in an intensive care unit, depending on their condition. The median ages of the study populations were 52 years [35], 67 years [37], 72 years [39], 62.5 years [40], 57 years [41], 58.5 years [42], and 61.7 years [43], with the majority of patients being male, as presented in Table 2 and Figure 2. Two studies, [36,38], did not report the median age of the study sample. The study population was primarily comprised of individuals who were of an older age. The proportion of patients who were admitted to an intensive care unit ranged from 6.7% to 34.3%, with the exception of two studies [38,40] that examined only patients who were hospitalized in an ICU and one study [43] that did not disclose the rate of ICU admissions.

According to the findings of one study [35], almost two-thirds of the patients who passed away were suffering from a secondary infection. The number of days that comprised the median stay in the hospital ranged from 7 to 24 days. There was a gap in the reporting of data concerning the length of stay in three of the trials. When it came to the mortality rate, the estimations of death were quite different, ranging from a 5.5 % to 38.7% of the total number of deaths. Table 2 contains both the demographic parameters and the clinical outcomes of the study population. When looking at other unfavorable outcomes, one study found that 10.6% of the severe SARS-CoV-2 cases required mechanical ventilation (MV) [36], another study found that 15% of the total cohort sample required MV [37], and three other studies found rates of 96% [41], 15.2% [42], and 14% [43].

At the time of admission, patients presented with a variety of clinical symptoms, including fever, cough, dyspnea, vomiting, and general malaise [39,42,43]. Diabetes [35,36,39,40,43], cardiovascular disease [35,36,40,43], chronic kidney disease [35,36,39], organ malignancy [36,39], and obesity [43] were the comorbidities that were seen the most frequently [35,36,39,40,43]. It was discovered that diabetes had the highest prevalence, with rates of 54.0%, 18.7%, 32.8%, 30.0%, 28.0%, and 18.3% [35,36,39,40,43]; the other diseases had lower rates. In addition, substantial rates of hypertension were discovered in two separate trials, with a prevalence of 66.8% [40], 48.0% [41], and 21.5% [43]. Concerning the seriousness of the COVID-19 diagnosis, one study [36] reported a prevalence of 19.3% [36] of severe cases included, whereas the other three studies [38,40,41] included only seriously ill patients who received medical assistance in the intensive care unit [36].

### 3.2. Bacterial Pathogen Detection

Every included study found *Staphylococcus aureus* to be present in the studied sample. Another pathogen that was found in most of the studies was *Klebsiella* spp. Regarding the prevalence of bacterial pathogens found in each study individually, one study found *Chlamydophila pneumonia* were the most prevalent, with a prevalence of 28.0% [35]. Another study found *S. aureus* (22.2%), *Pseudomonas aeruginosa* (27.8%), and *Klebsiella* spp. (25%) [36]. When differentiating between community-acquired infection and nosocomial infection, one study found *H. influenzae*, *S. pneumoniae*, *M. catarrhalis*, and *E. cloacae* to be the most prevalent for patients that had a community-acquired infection, and *P. aeruginosa* and *S. aureus* were the most prevalent for patients with nosocomial infection [37]. Another study that analyzed community-acquired infections found *Staphylococcus aureus* (27.0%), *Haemophilus infuenzae* (13.5%), *Streptococcus pneumoniae* (5.5%), *Moraxella catarrhalis* (2.5%), and *Legionella pneumophila* (1.5%) to be the most prevalent bacterial infection [42]. This high prevalence was in line with another study, which reported an occurrence of 59.7% bacterial pathogens, of which *Pseudomonas aeruginosa* (17.9%) and *Klebsiella pneumoniae* (13.4%) were the most frequently encountered [39]. In critically ill patients admitted to intensive care units, high rates of respiratory bacterial co-infection were found, comprising more than 30% of positive cultures, consisting, for the most part, of *Pseudomonas aeruginosa* (14.7%); *Klebsiella pneumoniae* (6.9%) and *Staphylococcus aureus* (6.5%) [38]; *Staphylococcus aureus* (21.7%); *Klebsiella pneumoniae* (20%) and *Haemophilus influenzae* (15%) [40]; and *P. aeruginosa*, *E. coli*, and *Klebsiella spp*. [41]. Lastly, a final study reported a co-infection in 40.8% of cases, with the most frequent bacteria being *Staphylococcus aureus* (11.8%), *Streptococcus agalactiae* (10.7%), *Haemophilus influenzae* (10.7%), and *Klebsiella pneumoniae* (8.6%) [43]. When differentiating between Gram-negative and Gram-positive bacteria, one study reported a frequency of 28% Gram-negative bacteria [35], while another reported a prevalence of 49.3% [39], as described in Table 3.

Only five of the studies [36,40,41,42,43] recorded the percentage of antibiotic use in the patient’s previous treatment before hospitalization. The rates were consistently high across the board, coming in at 40.4% [36], 73% [40], 79% [41], 51.5% [42], and 82% [43]. A few of the studies reported the usage of antibiotics such as cephalosporins, macrolides, and penicillin [36]; ampicillin and ceftriaxone [42]; and azithromycin [43]. When tested with mPCR (BioFire^®^ FilmArray^®^), patients who had taken antibiotics had a 13.7% chance of receiving a false-negative result, but patients who had been tested using a conventional bacterial culture had a 48% chance of receiving a false-negative result [36]. In a third study using a RT-qPCR assay, antibiotic-resistance genes were found in 14.2% of patients who had a bacterial infection [43].

A second study found that mPCR led to antibiotic changes—withdrawal, initiation, adaptation, de-escalation, or change because of inadequacy—therefore providing an optimal antibiotic management [41]. The BioFire^®^ FilmArray^®^ Pneumonia plus Panel was the most popular multiplex PCR, and it had the ability to detect 18 different bacteria, nine different viruses, and seven different antibiotic-resistance genes in under 1.5 h [41]. Although the majority of the studies did not report the sensitivity and specificity of the multiplex PCR on the study cohorts, there were four studies that did encounter a high sensitivity as well as a high specificity—78.4% [37], 89.6% [38], 89.3% [41], and 96.3% [19] for sensitivity and 98.1% [13], 98.3% [14], 99.1% [17], and 97.2% for specificity. One study reported a concordance of 55.6% between the multiplex PCR and microbiological culture [40]. Information about bacterial co-occurrence, use of antibiotics, type of multiplex PCR, and its accuracy in detecting bacterial infections are found in Table 3.

## 4. Discussion

This systematic review aimed to identify the assessment of multiplex PCR in detecting bacterial infections among patients hospitalized due to COVID-19. The main bacteria that found were *Klebsiella pneumoniae*, *S. aureus*, *Pseudomonas aeruginosa*, *Streptococcus pneumoniae*, and *Haemophilus influenzae.* These results are in line with other reviews that found *Streptococcus pneumoniae*, *S. aureus*, and *Klebsiella pneumoniae* [4,44] and *Pseudomonas aeruginosa* and *Haemophilus influenzae* [7] to be the most prevalent co-infections. As *S. pneumoniae* was found to be one of the most frequent pathogens, one review states that pneumococcal vaccination could decrease the burden of the COVID-19 virus, as it can prevent hospitalization and, thus, the risk of contracting nosocomial bacteria [45]. Despite the occurrence of *Mycoplasma pneumoniae* [4,9] reported in other systematic reviews and meta-analyses, studies included in the present review did not report a prevalence of this type of bacterium. In COVID-19 patients, other studies have reported a low co-occurrence of bacterial co-infections and superinfections [46,47]. However, the findings of this investigation demonstrated a significant incidence of respiratory bacterial co-infection, such as 30% [38] or even 40% [43]. This highlights a significant problem because the presence of bacterial infection can lead to unfavorable outcomes, such as an increased death rate [15]. As a result, the unfavorable outcomes reported in this study include the proportion of patients who required admission to an intensive care unit and the proportion of patients who passed away. Better treatment-management can be achieved using PCR as a diagnosis tool for bacterial identification and antibiotic resistance, given the high rates of mortality and bacterial co-infection among patients who need to be transferred into an intensive care unit.

When looking at techniques for the detection of pathogens, other studies have shown that the commercial multiplex RT-PCR approach has a high level of sensitivity (in the range of 95%) as well as specificity that reaches 99% [48,49]. Not only in the detection of viruses, in which the rates of detection have been proven to be higher, with positive predictive values higher than 80% [50], but also in the detection of bacterial infections, this finding highlights the importance of the mPCR in delivering an accurate detection in a timely and efficient manner [51]. One of the most significant issues that was discovered was the extensive use of antibiotics, which can result in unfavorable outcomes. This is due to the fact that extensive use of antibiotics can result in antibiotic resistance, which in turn will make the treatment plan more complicated [52]. Therefore, screening for bacterial co-infection becomes extremely important in order to identify the most effective methods of treatment that will not jeopardize the outcomes for the patient and, as a result, to reduce the use of antibiotics to the point where they are only used when absolutely necessary [30]. This calls for clinical judgment in approaching, in an effective manner, the treatment of COVID-19 infections, as well as other infections that may occur, by taking into account the potential for interactions between the infections and the medications that may be prescribed or the decision to admit patients to a hospital.

The strengths of this study include the range of locations in which the studies took place, with each study reporting information from a different country, in total covering Europe, South America, and Asia. This aspect is important because it creates a more accurate overview of the situation regarding bacterial infections and their outcomes in different parts of the globe. Secondly, the review only includes studies that detected pathogens by using the multiplex PCR or multiplex RT-PCR assays with different detection panels. Therefore, more accurate conclusions can be drawn since there were not disparate methods of testing that could potentially affect conclusions. One of the principal benefits of using multiplex PCR is the time waiting for detection, with a median of 5.5 h, which is much lower than that of standard culture, where the median is 26 h [49]. This is an important in light of the knowledge that we gained as a result of the recent pandemic, which taught us, among other things, that the treatment process, beginning with diagnosis, requires immediate attention [4]. Methods that suggest a fast detection of bacteria become significant in the management of critically ill patients [13]. This is because broad-spectrum antibiotics are often administered prior to collecting the microbiological data, making methods that provide a fast detection of infections essential to avoid false-negative results.

Another strength of this research is that, in contrast to other reviews, which focused mainly on the prevalence of bacterial co-infection [2,4], the current study was able to have an overview of clinical outcomes, as well as of bacterial co-infections in interaction with antibiotic usage and with the methods of testing. A further strength is that we were able to find a correlation between the use of antibiotics and the prevalence of bacterial co-infections. Naturally, this systematic review has a series of limitations. Firstly, both the number of included studies and the sample sizes of the studies themselves were relatively small, which means that generalizations should be approached with caution. Secondly, not all the included studies reported all the data that we sought to analyze. Although most of the studies had reported the necessary data when looking at ICU admissions and mortality rates, when it came to the characteristics of the conventional and RT multiplex PCR, in terms of accuracy, very few studies reported its specificity and sensitivity in the studied cohort. The same occurred with regard to the utilization of antibiotic treatment. In four of the papers, the rates of antibiotic use were not mentioned; rather, the focus was on the necessity of discouraging patients’ routine practice of taking medication even when it was unclear whether or not they required it. As a result, the remaining studies did not publish the percentage of severe cases, with the exception of one study that reported the proportions of COVID-19 grouped by the severity degree [36] and three studies that included only critically ill patients [38,40,41]. This is significant when one considers that patients who need intensive care have a greater prevalence of co-infection and, as a result, a higher rate of mortality [35,52].

Considering the risks that occur with antibiotic therapy in bacterial co-infection for patients infected with SARS-CoV-2, future studies should concentrate on gathering data regarding ways in which medication interacts with this specific group in order to find the most effective ways of treatment without compromising the patient’s recovery. One of the elements that might be taken into account is the individual’s current immunization status. We were unable to determine how vaccination interacts with this clinical picture since none of the trials that were included indicated the proportion of patients who were vaccinated or were not vaccinated. Although we could illustrate the most prevalent pathogens, the results are still quite disparate, as bacteria such as *Chlamydophila pneumonia* [35], *E. cloacae* [36], *E. coli* [41], and *Legionella pneumophila* [42] each only appeared in a singular study as being one of the most prevalent; thus, future research needs to continue to analyze different pathogens and their interactions by taking into account the studied sample.

## 5. Conclusions

Bacterial infections that are identified in COVID-19 patients can lead to more negative outcomes, particularly when it comes to patients with a severe infection who are hospitalized with COVID-19. Taking into account the high rates of co-infection and the high percentage of antibiotics used by individuals prior to hospitalization, effective methods of timely diagnosis and treatment need to be implemented in order to decrease the mortality rate and prevent the misdiagnosis of infections. This will allow for a reduction in the number of infections that are incorrectly diagnosed. Regarding the different PCR assays, both multiplex PCR and RT-PCR have high sensitivity and specificity, regardless of the panel used for bacterial detection, therefore making them ideal methods of detecting bacterial co-infections in COVID-19 patients.

## Figures and Tables

**Table 1:** Study characteristics.

Study	Country	Study Year	Study Design	Sample Size
1. [35] Alosaimi, B.; Naeem, A.; Hamed, M. E.; Alkadi, H. S.; Alanazi, T.; Al Rehily, S. S.; Almutairi, A. Z.; Zafar, A. Influenza Co-Infection Associated with Severity and Mortality in COVID-19 Patients. *Virology Journal* **2021**, *18* (1).	Saudi Arabia	2021	Retrospective observational	48
2. [36] Bogdan, I.; Citu, C.; Bratosin, F.; Malita, D.; Romosan, I.; Gurban, C. V.; Bota, A. V.; Turaiche, M.; Bratu, M. L.; Pilut, C. N.; Marincu, I. The Impact of Multiplex PCR in Diagnosing and Managing Bacterial Infections in COVID-19 Patients Self-Medicated with Antibiotics. *Antibiotics* **2022**, *11* (4), 437.	Romania	2022	Cross-sectional	489
3. [37] Cohen, R.; Babushkin, F.; Finn, T.; Geller, K.; Alexander, H.; Datnow, C.; Uda, M.; Shapiro, M.; Paikin, S.; Lellouche, J. High Rates of Bacterial Pulmonary Co-Infections and Superinfections Identified by Multiplex PCR among Critically Ill COVID-19 Patients. *Microorganisms* **2021**, *9* (12), 2483.	Israel	2021	Retrospective observational	93
4. [38] Foschi, C.; Zignoli, A.; Gaibani, P.; Vocale, C.; Rossini, G.; Lafratta, S.; Liberatore, A.; Turello, G.; Lazzarotto, T.; Ambretti, S. Respiratory Bacterial Co-Infections in Intensive Care Unit-Hospitalized COVID-19 Patients: Conventional Culture vs. BioFire FilmArray Pneumonia plus Panel. *Journal of Microbiological Methods* **2021**, *186*, 106259.	Italy	2021	Retrospective observational	178
5. [39] Huang, C.-P.; Tsai, C.-S.; Su, P.-L.; Huang, T.-H.; Ko, W.-C.; Lee, N.-Y. Respiratory Etiological Surveillance among Quarantined Patients with Suspected Lower Respiratory Tract Infection at a Medical Center in Southern Taiwan during COVID-19 Pandemic. *Journal of Microbiology*, *Immunology and Infection* **2022**, *55* (3), 428–435.	Taiwan	2022	Retrospective observational	201
6. [40] Karolyi, M.; Pawelka, E.; Hind, J.; Baumgartner, S.; Friese, E.; Hoepler, W.; Neuhold, S.; Omid, S.; Seitz, T.; Traugott, M. T.; Wenisch, C.; Zoufaly, A. Detection of Bacteria via Multiplex PCR in Respiratory Samples of Critically Ill COVID-19 Patients with Suspected HAP/VAP in the ICU. *Wiener klinische Wochenschrift* **2021**, *134* (9–10), 385–390.	Austria	2021	Retrospective observational	60
7. [41] Maataoui, N.; Chemali, L.; Patrier, J.; Tran Dinh, A.; Le Fèvre, L.; Lortat-Jacob, B.; Marzouk, M.; d’Humières, C.; Rondinaud, E.; Ruppé, E.; Montravers, P.; Timsit, J.-F.; Armand-Lefèvre, L. IMPACT OF RAPID Multiplex PCR on Management of Antibiotic Therapy in COVID-19-Positive Patients Hospitalized in Intensive Care Unit. *European Journal of Clinical Microbiology & Infectious Diseases* **2021**, *40* (10), 2227–2234.	France	2021	Retrospective observational	191
8. [42] Rothe, K.; Spinner, C. D.; Panning, M.; Pletz, M. W.; Rohde, G.; Rupp, J.; Witzenrath, M.; Erber, J.; Eberhardt, F.; Essig, A.; Schneider, J. Evaluation of a Multiplex PCR Screening Approach to Identify Community-Acquired Bacterial Co-Infections in COVID-19: A Multicenter Prospective Cohort Study of the German Competence Network of Community-Acquired Pneumonia (CAPNETZ). *Infection* **2021**, *49* (6), 1299–1306.	Germany and Switzerland	2021	Prospective observational multicenter	200
9. [43] Soto, A.; Quiñones-Laveriano, D. M.; Valdivia, F.; Juscamayta-López, E.; Azañero-Haro, J.; Chambi, L.; Horna, H.; Patiño, G.; Guzman, E.; De la Cruz-Vargas, J. A. Detection of Viral and Bacterial Respiratory Pathogens Identified by Molecular Methods in COVID-19 Hospitalized Patients and Its Impact on Mortality and Unfavorable Outcomes. *Infection and Drug Resistance* **2021**, *Volume 14*, 2795–2807.	Peru	2021	Prospective observational	93

**Table 2:** Demographic data and clinical outcomes.

Study	Age (Median)	Gender (% Female–Male)	ICU Admissions (%)	Duration of Hospitalization (Median)	Mortality Rate (%)
Alosaimi [35]	52.0	33.0–77.0%	29.0	ND	19.0
Bogdan [36]	>18.0	49.0–51.0%	6.7	12.4	5.5
Cohen [37]	67.0	30.0–70.0%	ND	24.0	38.7
Foschi [38]	>18.0	ND	100	ND	ND
Huang [39]	72.0	31.8–68.2%	9.5	7.0	8.5
Karolyi [40]	62.5	20.0–80.0%	100	7.0	36.7
Maataoui [41]	57.0	18.0–82.0%	100	19.0	56.0
Rothe [42]	58.5	36.5–63.5%	23.4	9.0	4.5
Soto [43]	61.7	29.0–71.0%	ND	ND	31.0

ND (no data reported).

**Table 3:** Bacterial pathogens involved.

Study	Most Frequent Bacteria	Community-Acquired	Antibiotic Usage Prior to Admission (%)	PCR Assay and Panels	Sensitivity/Specificity (%)
1 [35]	*Chlamydophila pneumonia*, *S. aureus*	ND	ND	Multiplex PCR assay, Superscript III panel	ND
2 [36]	*Staphylococcus aureus*, *Pseudomonas aeruginosa*, *Klebsiella* spp.	25.5%	40.4	Multiplex RT-PCR assay	ND
3 [37]	*H. influenzae*, *S. pneumoniae*, *M. catarrhalis*, *E. cloacae*, *P. aeruginosa*, *S. aureus*	17.0% (31.5% *K. pneumoniae*, 17.3% *H. influenzae*, *S. pneumoniae* 13.0%)	ND	Biofire^®^, FilmArray^®^ Pneumonia Panel	78.4/98.1
4 [38]	*Pseudomonas aeruginosa*, *Klebsiella pneumoniae*, *Staphylococcus aureus*	ND	ND	FilmArray Pneumonia Plus panel	89.6/98.3
5 [39]	*Pseudomonas aeruginosa*, *Klebsiella pneumoniae*, *Staphylococcus aureus*	23.4%	ND	FilmArray TM Respiratory Panel	ND
6 [40]	*Staphylococcus aureus*, *Klebsiella pneumoniae*, *H. influenzae*	0.0% (20% hospital-acquired, 80% ventilator-acquired)	73.0	BioFire^®^ Pneumonia Panel	ND
7 [41]	*P. aeruginosa*, *E. coli*, *Klebsiella* spp.	ND	79.0	BioFire^®^ FilmArray^®^ Pneumonia plus Panel	89.3/99.1
8 [42]	*Staphylococcus aureus*, *H. influenzae*, *Streptococcus pneumoniae*, *Moraxella catarrhalis*, *Legionella pneumophila*	100% (*S. aureus* 27.0%, *H. influenzae* 13.5%, *S. pneumoniae* 5.5%)	51.5	Multiplex RT-PCR	ND
9 [43]	*Staphylococcus aureus*, *Streptococcus agalactiae*, *H. influenzae*, *Klebsiella pneumoniae*	ND	82.0	Biofire Filmarray Pneumonia plus^®^ panel	96.3/97.2

ND (no data reported).

## Data Availability

Not applicable.
